# Optimizing discharge outcomes in very preterm infants by a novel integrated family and rehabilitation care model—a retrospective case-matched study

**DOI:** 10.3389/fped.2026.1867308

**Published:** 2026-06-24

**Authors:** Ren Zhuxiao, Ren Jianbing, Yi Aiwen, Mo Shisheng, Wang Zhu, Yang Shumei, Yin Shiyao, Nie Chuan

**Affiliations:** 1 Department of Neonatology, Guangdong Women and Children Hospital, Southern University of Science and Technology, Guangzhou, China; 2 Guangdong Neonatal ICU Medical Quality Control Center, National Key Clinical Specialty Construction Unit, Guangzhou Medical University, Guangzhou, China; 3 Department of Rehabilitation Medicine, Guangdong Women and Children Hospital, Southern University of Science and Technology, Guangzhou, China

**Keywords:** case-matched study, discharge outcomes, integrated family and rehabilitation care, retrospective, very preterm infants

## Abstract

**Background:**

In our routine NICU settings, traditionally implemented family-centered care (FCC) often showed limitations including insufficient parental involvement, high caregiver burden, and lack of systematic rehabilitation integration, which restricted improvements in respiratory, feeding, and neurodevelopmental outcomes. This study introduced a novel integrated family-centered care (NIFCC) model combining daytime family-integrated care, bedside rehabilitation, and nighttime professional NICU care, and investigated its effect on discharge outcomes in very preterm infants compared with traditionally implemented FCC in our clinical setting.

**Methods:**

A retrospective 1:1 case-matched study was conducted in a level 4 NICU. Fifty-three very preterm infants (gestational age <32 weeks) receiving NIFCC were 1:1 matched with 53 controls under our traditionally implemented family-centered care. Primary outcomes included oxygen weaning, full oral feeding establishment, and Neonatal Behavioral Observation (NBO) scores. Secondary outcomes included complications, anthropometric measures, hospital stay, and costs.

**Results:**

The NIFCC group had significantly higher oxygen independence (92.5% vs. 73.6%, *P* = 0.018) and shorter oxygen therapy (18 vs. 25 days, *P* = 0.019). Full oral feeding rate was 100% vs. 86.8% (*P* = 0.013), and NBO scores were higher (51 vs. 49, *P* = 0.036). Discharge anthropometrics were superior. No significant differences in preterm complications, hospital stay, or costs were found, and no adverse events occurred.

**Conclusion:**

The NIFCC model is safe and effective, significantly improving oxygen weaning, oral feeding, neurobehavioral development, and growth in very preterm infants. By addressing the incomplete implementation of traditional FCC caused by insufficient staff training and lack of care culture transformation, NIFCC provides a valuable strategy for optimizing NICU care quality.

## Introduction

Very preterm infants (VPIs, gestational age <32 weeks) are a highly vulnerable population with high risks of respiratory insufficiency, feeding immaturity, neurodevelopmental delay, and adverse long-term outcomes (1, 2). Advances in perinatal and neonatal intensive care have greatly improved survival rates, yet optimizing discharge readiness and neurodevelopmental outcomes remains a major clinical challenge in neonatal intensive care units (NICUs) worldwide (3, 4). Respiratory weaning, establishment of full oral feeding, and early neurobehavioral maturation are critical determinants of hospital discharge timing, length of stay, costs, and long-term prognosis for these infants (5, 6). Therefore, developing safe, effective, and sustainable care models to improve these core outcomes has become a priority in neonatal care research and practice.

Family participation and developmental supportive care are increasingly recognized as essential components of high-quality NICU care (7, 8). Traditional care models have long focused on medical stabilization and technical support, with limited family involvement and insufficient integration of early rehabilitation interventions (9, 10). In recent years, family-centered care (FCC) has emerged as a paradigm shift in neonatal care, emphasizing partnership between families and healthcare providers to promote infant development and family well-being (11, 12). Various FCC-based models have been explored, including standard family-centered care, family integrated care (FICare), and 24-h family rooming-in (13, 14). However, real-world implementation of these models is often incomplete due to insufficient staff training, inconsistent care culture, high parental burden, and lack of structured rehabilitation components (5, 15, 16).

In our center, parents often acted as observers rather than active care providers, resulting in insufficient improvement in family care capabilities after discharge (5). While, FICare, which has been widely promoted globally, allows parents to become formal members of the care team and participate in daily neonatal care, significantly improving feeding success rates and reducing hospital stays; however, it requires long-term parental accompaniment, thus potentially leading to high physical and mental burden, fatigue, and even care burnout among parents (6). The 24-h full family rooming-in model maximizes parent-infant contact and provides optimal developmental support, but the 24-h care responsibility places extreme pressure on parents, and the lack of professional nighttime supervision may increase potential safety risks for neonates (1–3). In addition, traditional family-centered care models generally lack systematic integration with rehabilitation interventions, making it difficult to provide continuous and targeted support for neonatal respiratory function, oral feeding, and neurodevelopment—three core indicators that determine the timing of discharge and long-term prognosis (7).

To address the incomplete implementation and limitations of traditionally implemented FCC in our center, we proposed a novel integrated family-centered care model, which combines daytime FICare with single-family rooms, bedside rehabilitation guidance by physical therapists, and nighttime return to NICU for professional care. This new model was implemented in our NICU since 2024. Recent studies have shown that single-family rooms in NICUs can enhance neonatal outcomes, reduce sepsis rates, and promote better weight gain, although challenges such as isolation and reduced sensory stimulation for infants persist. Furthermore, nursing care in NICUs has proven essential in mitigating stressors and optimizing developmental trajectories, ensuring better sleep quality and overall well-being for vulnerable neonates (8). This new model retains the advantages of traditional FICare, such as high parental participation and improved discharge preparation, while overcoming its shortcomings by providing professional nighttime care to ensure adequate parental rest and avoid care burnout. Meanwhile, the integration of bedside rehabilitation interventions directly targets neonatal respiratory function, oral motor function, and neurodevelopment, forming a multi-dimensional care system that balances medical safety, family participation, and neonatal developmental needs (9).

This integrated model holds theoretical potential to promote timely oxygen weaning, full oral feeding, and optimal neurodevelopment before discharge—key outcomes closely related to survival quality and long-term prognosis of very preterm infants. We therefore designed this study to assess whether the novel integrated family-centered care model improves short-term developmental outcomes compared with conventional care, with the aim of generating evidence to support its clinical application.

## Methods

### Study design and population

The neonatal intensive care unit (NICU) at Guangdong Women and Children Hospital in Guangzhou, Guangdong, China, is a level 4, 140-bed unit, primarily serving inborn infants, with around 5,000 admissions per year. A retrospective 1:1 case-match observational study was conducted in the neonatal intensive care unit (NICU). Participants were preterm infants with a gestational age <32 weeks admitted between July 2024 and December 2025.

### Inclusion criteria

Infants fulfilling all the following inclusion criteria were enrolled in this trial: (1) birth at a study hospital; (2) gestational age at birth <32 weeks (GA was calculated based on the date of the last menstrual period of the mother and an ultrasonographic screening performed during the first trimester of pregnancy); (3) free of severe perinatal asphyxia (defined as an Apgar score of 0–3 for more than 5 min, a cord blood gas pH <7.00, or both); (4) free of severe congenital anomalies or genetic syndromes; (5) Presence of any one of the following: oral feeding difficulty or weaning difficulty from supplemental oxygen after GA being >34 weeks; (6) with complete medical records and discharge data.

### Exclusion criteria

Infants were excluded from the study if: (1) death or transfer to another hospital during hospitalization; (2) incomplete clinical data; (3) parents with severe physical or psychiatric illness preventing care participation.

### Matching and grouping

A 1:1 matched case-control design was used. Infants in the study group receiving the integrated family care model were matched with control infants at a 1:1 ratio according to: gestational age, birth weight, gender, 1.5-min Apgar score, and level of respiratory support after birth. After stabilization, the infant in the study group was transferred to a family-centered ward. The criteria of stabilization were no requirement for non-invasive or invasive respiratory support; no severe acute infection (e.g., sepsis, pneumonia, meningitis); no clinical seizures or requirement for anticonvulsant therapy; no requirement for continuous intravenous medication other than standard parenteral nutrition. The physicians would talk with the parents, if they were able and willing to engage in our novel family-integrated care, they were classified as study group; otherwise were classified as control group. The study group received the integrated family-centered care model. The control group received conventional NICU care and traditional family-centered care model without structured parental involvement and systematic bedside rehabilitation.

### Study procedures

Infants in the study group received the (NIFCC) model, which included three core components: enhanced parental participation in daytime family-integrated care (FICare), individualized bedside rehabilitation therapy, and nighttime professional safety care in the NICU. The specific interventions were detailed as follows:

1. Daytime Family-Integrated Care (FICare) with Enhanced Parental Participation

Parents completed standardized training before involvement, including hygiene, basic care, safe handling, recognition of infant cues, skin-to-skin care, feeding assistance, and emotional support. Under direct supervision of nurses and physical therapists, parents performed daily care including diaper care, positioning, soothing, oral feeding support, and parent-infant interaction. Individualized education and psychological support were provided daily to reduce parental anxiety and improve care competence.

2. Bedside Rehabilitation Interventions

A dedicated physical therapist provided structured bedside rehabilitation once a day 5–6 days per week. For infants with feeding abnormalities, an additional specialized swallowing function assessment was performed.

Rehabilitation therapy was conducted daily in the rehabilitation therapy room in NICU, with each session lasting approximately 30 min. Based on the assessment results, the therapist developed an individualized rehabilitation plan for each infant, which may include, but was not limited to behavioral organization and state regulation (including developmental positioning and respiratory support and activity tolerance training); passive and active range of motion exercises, head control training and motor facilitation; sensory-motor interventions (including deep pressure, graded touch, vestibular stimulation, musculoskeletal rehabilitation, stretching, joint approximation) and targeted training for swallowing dysfunction-oral sensory and oral motor training to improve sucking-swallowing-breathing coordination.

The treatment environment was strictly controlled for comfort and safety, with appropriate room temperature and humidity maintained, and soft, soothing background music played to create a warm and relaxed therapeutic atmosphere. During treatment, the therapist firstly showed standardized procedures, followed by one-on-one guidance to help parents master core techniques and perform basic rehabilitation exercises for their infants. The therapy room was equipped with specialized infant rehabilitation aids (auditory stimulation rattles, visual development black-and-white cards, tactile perception toys). When the infant was awake and calm, the therapist guided parents in parent-child interactive games to promote neurodevelopment through multi-sensory stimulation. Parents may provide their own massage oil, and the therapist instructed them on standardized neonatal full-body massage techniques to strengthen the parent-infant bond and improve neurobehavioral development and physical comfort through tactile stimulation.

3. Nighttime Safety Care

At night (20:00–08:00 the next day), infants were transferred back to the NICU from the family care unit, where professional nurses provided continuous monitoring, including: Continuous cardiorespiratory monitoring (including heart rate, blood oxygen saturation, respiratory rate), routine nursing care (such as temperature monitoring, diaper change, feeding if needed) and timely handling of abnormal conditions (such as desaturation, apnea, and bradycardia).

Regular handover with daytime medical and rehabilitation teams to ensure continuity of care, including reporting the infant's daytime care status, feeding situation, and rehabilitation progress to the nighttime nursing team.

### Traditional family-centered care (FCC, control group)

#### Control group (traditionally implemented family-centered care, FCC)

Traditionally implemented FCC in our NICU refers to the routine real-world practice of FCC before systematic staff training and care culture transformation. Family participation was limited to regular visitation (twice weekly, 30 min each time) mainly in the final week before discharge, without structured parental involvement in daily care or systematic bedside rehabilitation interventions. Parents were only allowed to observe the infant or have brief skin-to-skin contact under supervision, but could not perform independent care operations. This model reflects the incomplete implementation of FCC due to insufficient staff training and untransformed care culture, rather than a deviation from the FCC concept. Infants remained in the NICU throughout hospitalization, and all daily care was completed by professional nurses.

### Outcomes

#### Primary outcomes

1. Proportion of infants who achieved complete oxygen independence (no need for any supplemental oxygen) before discharge and the time to discontinuation of oxygen therapy (from the start of mechanical ventilation to complete oxygen withdrawal); 2. Proportion of infants who achieved full oral feeding (no need for tube feeding, able to meet full nutritional needs through oral feeding) before discharge and the time from the first oral feeding to full oral feeding; 3. Neonatal neurobehavioral assessment using the Neonatal Behavioral Observation scores (NBO) by physical therapists at discharge. The Neonatal Behavioral Observation (NBO) scale was used to assess neonatal neurobehavioral development at discharge. The NBO is a well-established, validated tool with strong reliability and validity for evaluating neurobehavioral function in preterm infants. It comprises 18 key items covering habituation, motor performance, social interaction, and stress response, with established psychometric properties supporting its clinical utility. Furthermore, the NBO has been widely documented to correlate well with gold-standard developmental assessments such as the Bayley Scales of Infant Development (BSID-III) and is globally integrated into neonatal care and research (10).

#### Secondary outcomes

1. Incidence of common preterm complications including BPD, intraventricular hemorrhage (IVH), necrotizing enterocolitis (NEC), retinopathy of prematurity (ROP), and LOS; 2.Anthropometric outcomes including weight, height and head circumference; 3. Length of hospital stay: total duration from admission to discharge (days); 4. Total hospitalization cost.

### Sample size

Sample size was calculated with Stata version 17.0 (StataCorp) based on the primary outcome of successful oxygen weaning before discharge. Assuming a success rate of 50% in the control group and 80% in the intervention group, with a two-sided type I error *α* = 0.05 and a power of 80%, a minimum of 45 infants per group was required. Considering a 15% dropout rate, at least 52 infants per group were planned. 1:1 exact matching was performed using the MatchIt package in R software (version 4.3.1). The intervention group was used as the reference, and matching covariates included gestational age, birth weight, gender, 1-min Apgar score, 5-min Apgar score, and level of respiratory support after birth. Exact matching without replacement was applied. Each infant in the intervention group was matched to one control infant with the most similar baseline characteristics. Thus, a total of 106 infants (53 matched pairs) were included in this study.

### Statistical analysis

All statistical analyses were performed using SPSS 26.0 (IBM Corp., Armonk, NY, USA). Categorical variables were summarized as frequencies and percentages, and compared using the McNemar test for paired data. Continuous variables were tested for normality. Normally distributed data were presented as mean ± standard deviation and compared using the paired *t*-test. Non-normally distributed data were presented as median (interquartile range) and compared using the Wilcoxon signed-rank test. All tests were two-sided, and *P* < 0.05 was considered statistically significant.

### Ethical approval

This study was approved by the Ethics Committee of Guangdong Women and Children Hospital (202301194). All data were anonymized, and informed consent was waived due to the retrospective design.

### Role of the funding source

The funder of the study had no role in study design, data collection, data analysis, data interpretation, or writing of the report. No authors have been paid to write this article by a pharmaceutical company or other agency. Authors were not precluded from accessing data in the study, and they accept responsibility to submit for publication. Nie Chuan and Ren Zhuxiao had access to the dataset and had final decision to submit for publication.

## Results

### Baseline characteristics

**Figure 1:**
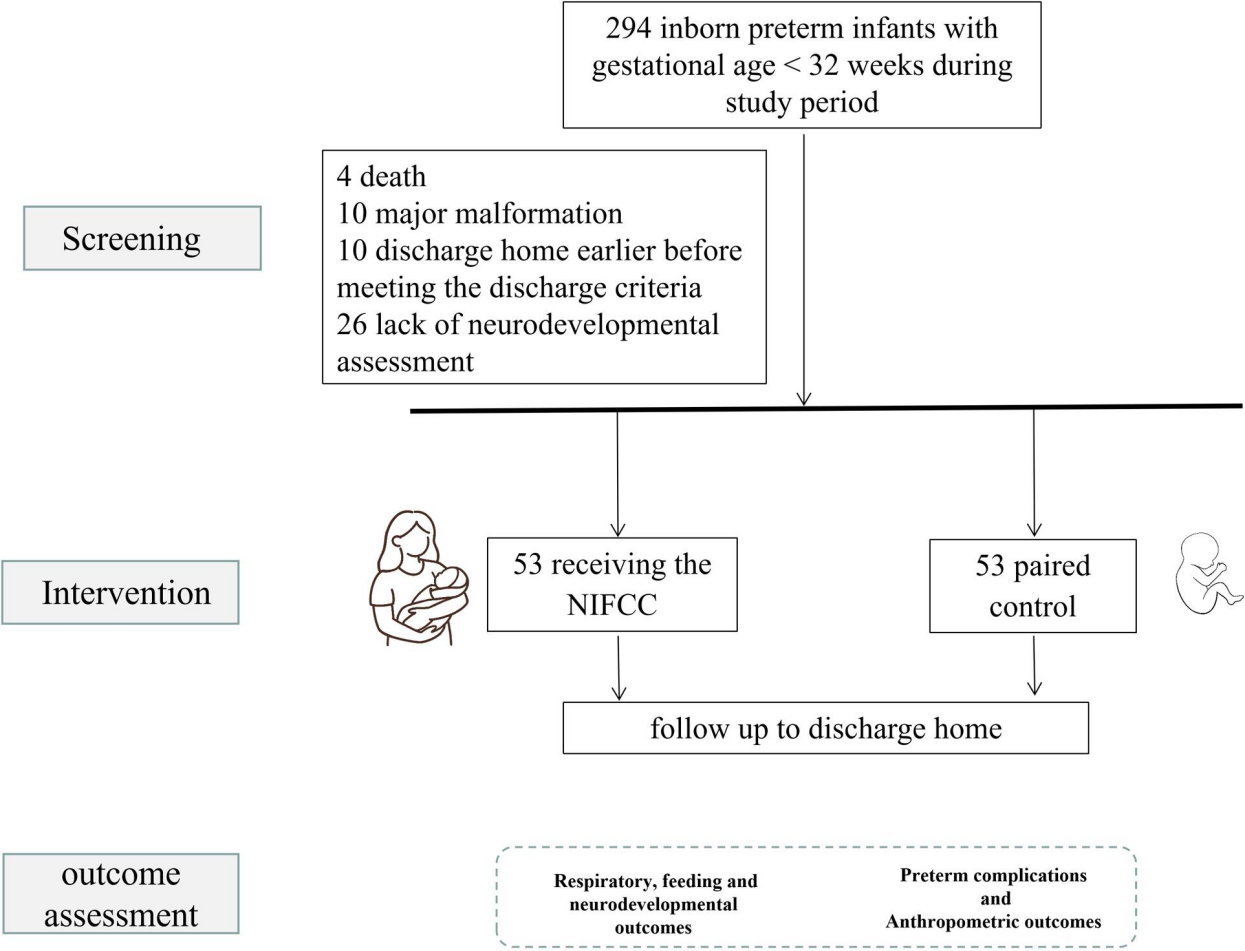
The design and process of this study.

Between July 2024 and December 2025, a total of 294 inborn preterm infants with gestational age <32 weeks admitted to the NICU of Guangdong Women and Children Hospital were screened for eligibility. Among them, 50 infants were excluded because of death (4), major malformation (10), discharge home earlier before meeting the discharge criteria (10 still needing non-invasive ventilation), and lack of neurodevelopmental assessment (26). Among the remaining 244 infants, 53 infants receiving the novel integrated family-centered care (NIFCC) and were enrolled in the study group. Among 181 infants in the control group, 53 were paired to the study group by 1:1 by GA, birth weight, gender, 1,5 min apgar score, and respiratory condition at birth. Each infant in the intervention group was matched to one control infant with the most similar baseline characteristics from 181 candidates. Finally, 53 matched pairs were included in the analysis. 106 infants were finally enrolled in the study, with 53 in the NIFCC group and 53 paired infants in the control group (receiving traditional family-centered care and NICU care) (Figure 1).

The main baseline characteristics of the mothers and infants were similar between the study group and the control group (Table 1). Matching criteria including gestational age, birth weight, gender, 1 and 5-min Apgar score, and level of respiratory support after birth showed no significant differences between the two groups (all *P* > 0.05). In addition, maternal age, height, weight, education level, model of delivery, presence of pregnancy complications and use of antenatal glucocorticoids were also comparable between the two groups (all *P* > 0.05). Infants condition at birth and swallowing function score before oral feeding also showed no significant difference in the two groups. All enrolled infants were followed up until their first discharge home, with no loss to follow-up.

**Table 1:** Baseline characteristics and status of the infants and their mothers in the two groups.

Characteristics	Control group (*N* = 53)	Intervention group (*N* = 53)	*P* Value
Mothers			
Age (years), median, IQR	32 (30–35)	33 (30–36)	0.685
Education, high school or junior, *n*, %	37 (69.8%)	27 (50.9%)	0.073
Antenatal glucocorticoids, *n*, %	29 (54.7%)	23 (43.4%)	0.331
Preeclampsia, *n*, %	4 (7.5%)	4 (7.5%)	1.000
Gestational hypertension, *n*, %	3 (5.7%)	5 (9.4%)	0.716
Gestational diabetes, *n*, %	12 (22.6%)	12 (22.6%)	1.000
Membrane rupture, *n*, %	13 (24.5%)	23 (43.4%)	0.064
Height (cm), mean ± SD	157.4 ± 5.2	157.7 ± 5.7	0.789
Weight before delivery (kg), mean ± SD	64.3 ± 9.3	64.7 ± 11.1	0.861
Infants at birth			
CS	40 (75.5%)	37 (69.8%)	0.663
Apgar Score			
1 min, median, IQR	8 (8–8)	8 (8–8)	1.000
5 min, median, IQR	9 (9–9)	9 (9–9)	1.000
10 min, median, IQR	9 (9–9)	9 (9–9)	1.000
GA (weeks), mean ± SD	29.9 ± 1.5	29.6 ± 1.5	0.231
Male, *n*, %	29 (54.7%)	30 (56.6%)	1.000
Birth height (cm), mean ± SD	36.3 ± 2.8	36.9 ± 3.5	0.394
Head circumference (cm), mean ± SD	27.1 ± 1.7	26.8 ± 2.0	0.269
Birth weight (kg), mean ± SD	1.31 ± 0.29	1.27 ± 0.26	0.553
Respiratory condition at birth			
Intubated, n, (%)	4 (7.5%)	10 (18.9%)	0.150
PS Treatment, *n*, (%)	37 (69.8%)	31 (58.5%)	0.311
RDS grade, median, IQR	2 (0–2)	1 (1–2)	0.581
EOS, *n*, (%)	16 (30.2%)	18 (34.0%)	0.835
Swallowing function score before Oral feeding, median, IQR	29 (28.5–30)	29 (29–30)	0.877

CS, caesarean section; GA, gestational age. Median and interquartile range (IQR) and paired non-parametric analysis was used for data with non-normal distribution. Mean and standard deviation (SD) and paired *t*-test was used for data with normal distribution.

### Study intervention

All infants in the study group completed the NIFCC model intervention as planned, including enhanced daytime FICare, individualized bedside rehabilitation therapy, and nighttime professional safety care in the NICU. The compliance rate of parental participation in daytime care was 100%, and the completion rate of bedside rehabilitation therapy (at least one of the five rehabilitation therapy) was 100% (Table 2). The median number of bedside rehabilitation sessions per infant was 5 [IQR = 3–9] sessions.

**Table 2:** Short term outcomes and rehabilitation intervention.

Outcomes	Control (*n* = 53)	Intervention (*n* = 53)	*P*
Primary outcomes			
Oxygen weaning before discharge, *n*, (%)	39 (73.6%)	49 (92.5%)	0.018
Duration of assisted oxygen therapy (day), median, IQR	25 (12.5–38.8)	18 (10–29.5)	0.019
Full oral feeding before discharge, *n*, (%)	46 (86.8%)	53 (100%)	0.013
Full oral feeding establishment duration (day), median, IQR	24 (17–38.2)	23 (20–31)	0.640
Neonatal Behavioral Observation scores, median, IQR	49 (47–51)	51 (49–53)	0.036
Secondary outcomes			
Height at 36 GW (cm), mean ± SD	40.61 ± 2.12	41.75 ± 2.50	0.013
Head circumference at 36 GW (cm), mean ± SD	29.82 ± 1.26	29.87 ± 1.52	0.868
Weight at 36 GW (kg), mean ± SD	2.01 ± 0.30	2.06 ± 0.33	0.364
Height before discharge (cm), mean ± SD	42.40 ± 2.0	45.25 ± 2.37	<0.001
Head circumference before discharge (cm), mean ± SD	31.21 ± 2.13	33.00 ± 1.52	0.030
Weight before discharge (kg), mean ± SD	2.34 ± 0.33	2.49 ± 0.28	0.017
BPD, *n*, (%)	23 (43.4%)	21 (39.6%,)	0.844
IVH, *n*, (%)	9 (16.4%)	7 (10.1%)	0.255
NEC, *n*, (%)	11 (20.8%)	4 (1.5%)	0.092
ROP, *n*, (%)	17 (32.1%)	23 (43.4%)	0.316
LOS, *n*, (%)	4 (7.5%)	3 (5.7%)	0.432
Pulmonary hemorrhage, *n*, (%)	2 (3.8%)	4 (7.5%)	0.678
Septic Shock, *n*, (%)	6 (11.3%)	5 (9.4%)	1.000
Duration of Hospitalization (day), mean ± SD	57 ± 20	53 ± 21	0.214
Hospitalization expense (RMB), mean ± SD	98,962 ± 62,722	85,450 ± 70,258	0.231
Rehabilitation therapy			
Head control training and motor facilitation, *n*, (%)	0 (0%)	12 (22.6%)	<0.001
Behavioral organization and state regulation, *n*, (%)	2 (3.8%)	48 (90.6%)	<0.001
Sensory-motor interventions, *n*, (%)	10 (18.9%)	50 (94.3%)	<0.001
Passive and active range of motion exercises, *n*, (%)	31 (58.5%)	53 (100%)	<0.001
Targeted training for swallowing dysfunction, *n*, (%)	42(80.8%)	49(92.5%)	0.092

GW, gestational weeks; BPD, bronchopulmonary dysplasia; IVH, intraventricular hemorrhage, NEC, necrotizing enterocolitis; ROP, retinopathy of prematurity; LOS, late onset sepsis. Median and interquartile range (IQR) and paired non-parametric analysis was used for data with non-normal distribution. Mean and standard deviation (SD) and paired *t*-test was used for data with normal distribution.

In the control group, all infants received traditionally implemented FCC as planned, with family participation limited to regular visits (2 times per week, 30 min each time) only in the last week before discharge, without active involvement in daily care or rehabilitation.

All infants in both groups received assisted oxygen therapy during hospitalization, and there was no significant difference in the initial level of respiratory support between the two groups (*P* > 0.05) (Table 1). No adverse events related to the NIFCC model were observed during the intervention period, including no neonatal safety incidents caused by parental care operations, no parental care burnout, and no adverse reactions related to bedside rehabilitation therapy.

### Outcomes

#### Primary outcomes

Statistically significant differences were observed in the primary outcomes between the two groups, with the study group showing significantly better outcomes than the control group (Table 2).

#### Respiratory outcomes

The proportion of infants who achieved complete oxygen independence before discharge in the study group reached 92.5% (49/53), which was significantly higher than 73.6% (39/53) in the control group (*P* = 0.018). The median duration of assisted oxygen therapy in the study group was 18 days (IQR = 10–29.5 days), significantly shorter than 25 days (IQR = 12.5–38.8 days) in the control group (*P* = 0.019).

### Full oral feeding establishment

The proportion of infants who achieved full oral feeding before discharge in the study group was 100% (53/53), significantly higher than 86.8% (46/53) in the control group (*P* = 0.013). There was no significant difference in the duration of full oral feeding establishment between the two groups (*P* = 0.640), with a median of 23 days (IQR = 20–31 days) in the study group and 24 days (IQR = 17–38.2 days) in the control group.

### Neonatal neurobehavioral assessment

The median Neonatal Behavioral Observation (NBO) score in the study group was 51 (IQR = 49–53), which was significantly higher than 49 (IQR = 47–51) in the control group (*P* = 0.036), suggesting a better neurobehavioral development status in the study group.

### Secondary outcomes

#### Anthropometric outcomes

At 36 weeks of corrected gestational age, the mean height of infants in the study group was 41.75 cm (SD = 2.5 cm), significantly higher than 40.61 cm (SD = 2.12 cm) in the control group (*P* = 0.013), while no significant differences were found in head circumference [29.87 cm (SD = 1.52 cm) vs. 29.82 cm (SD = 1.26 cm), *P* = 0.868] and body weight [2.06 kg (SD = 0.33 kg) vs. 2.01 kg (SD = 0.30 kg), *P* = 0.364] between the two groups. Before discharge, all anthropometric indicators of infants in the study group were significantly superior to those in the control group (all *P* < 0.05): the mean height was 45.25 cm (SD = 2.37 cm) vs. 42.40 cm (SD = 2.0 cm), the mean head circumference was 33.0 cm (SD = 1.52 cm) vs. 31.21 cm (SD = 2.13 cm), and the mean body weight was 2.49 kg (SD = 0.28 kg) vs. 2.34 kg (SD = 0.33 kg).

### Incidence of preterm complications

There were no statistically significant differences in the incidence of major preterm complications between the two groups. The incidence of bronchopulmonary dysplasia (BPD) was 39.6% (21/53) in the study group and 43.4% (23/53) in the control group (*P* = 0.844); intraventricular hemorrhage (IVH) was 10.1% (7/53) vs. 16.4% (9/53) (*P* = 0.255); necrotizing enterocolitis (NEC) was 1.5% (4/53) vs. 20.8% (11/53) (*P* = 0.092); retinopathy of prematurity (ROP) was 43.4% (23/53) vs. 32.1% (17/53) (*P* = 0.316); late-onset sepsis (LOS) was 5.7% (3/53) vs. 7.5% (4/53) (*P* = 0.432). In addition, the incidence of pulmonary hemorrhage (7.5% vs. 3.8%, *P* = 0.678) and septic shock (9.4% vs. 11.3%, *P* = 1.000) also showed no inter-group differences.

### Hospitalization-related indicators

Although patients in the intervention group needed less hospitalization duration and expense, there was no significant difference in the duration of hospitalization between the two groups (53 ± 21 days vs. 57 ± 20 days, *P* = 0.214), nor in the total hospitalization expense (85,450 ± 70,258 RMB vs. 98,962 ± 62,722 RMB, *P* = 0.231).

### Rehabilitation therapy implementation

The implementation rate of various rehabilitation therapy measures in the study group was significantly higher than that in the control group (all *P* < 0.001). Head control training and motor facilitation were implemented in 22.6% (12/53) of infants in the study group, with 0% in the control group. Behavioral organization and state regulation were applied in 90.6% (48/53) of the study group vs. 3.8% (2/53) of the control group. Sensory-motor interventions were conducted in 94.3% (50/53) of the study group vs. 18.9% (10/53) of the control group. Passive and active range of motion exercises were completed in 100% (53/53) of the study group vs. 58.5% (31/53) of the control group.

Targeted training for swallowing dysfunction was implemented in 92.5% (49/53) of the study group and 80.8% (42/53) of the control group, with no significant difference between the two groups (*P* = 0.092).

## Discussion

In the present study, we evaluated the effectiveness of NIFCC compared with traditionally implemented FCC in very preterm infants. The results showed that NIFCC significantly improved respiratory, feeding, neurobehavioral and growth outcomes without increasing adverse events, supporting its safety and clinical value.

A key issue addressed in this study is that traditionally implemented FCC in our center was incomplete, with limited parental participation and absence of systematic bedside rehabilitation. As suggested by related literature, this is not a failure of the FCC concept, but results from insufficient staff training in FCC and incomplete transformation of care culture (5). In most conventional NICUs, FCC was limited to basic communication and restricted visitation, without structured family involvement and integrated rehabilitation (17, 18). The NIFCC model overcomes the incomplete implementation of traditionally implemented FCC through three innovations. First, it enhances structured parental participation during daytime under professional guidance. Second, it systematically embeds individualized bedside rehabilitation into daily care, which was lacking in traditional FCC practice. Third, nighttime professional NICU care ensures safety and avoids parental burnout.

Notably, the lack of systematic rehabilitation in the control group was not due to differential performance of medical staff, but to historical care protocols, limited rehabilitation resources, and insufficient FCC training and untransformed care culture. We confirm that the same clinical team was responsible for both groups, and only the care model and rehabilitation protocol differed, which reduces potential performance bias in the analysis. As emphasized by previous studies, training NICU staff and transforming care culture are essential to achieve high-quality and full-implementation FCC (5). The NIFCC model provides a feasible pathway to realize this goal, making it an optimized strategy for preterm infant care in NICUs.

Another important innovation was the systematic incorporation of bedside rehabilitation interventions into routine family-centered care. Traditional family-centered programs rarely integrated targeted rehabilitation support for respiratory function, oral motor coordination, and neurodevelopment-core domains that directly determined discharge readiness and long-term prognosis (19). In this study, dedicated physical therapists performed daily individualized bedside rehabilitation, including behavioral state regulation, motor facilitation, respiratory tolerance training, oral sensory-motor therapy, and multi-sensory stimulation. By embedding rehabilitation into daily care and training parents to participate in basic therapeutic activities, the NIFCC model created a continuous, development-supportive care environment. This multi-dimensional intervention directly targeted the main challenges of delayed oxygen weaning and feeding immaturity in preterm infants (20). Such a comprehensive, family-engaged rehabilitation component distinguished the present model from conventional FICare and family rooming-in programs.

The NIFCC model also showed a positive promoting effect on the anthropometric growth of very preterm infants. The results showed that the height of the study group at 36 weeks of corrected gestational age and all anthropometric indicators (height, head circumference, body weight) before discharge are significantly superior to those of the control group. This might be because the NIFCC model created a stable and development-supportive care environment for preterm infants—the daytime parent-infant interaction and sensory stimulation promote the neonatal appetite and feeding compliance, while the systematic rehabilitation intervention improves the neonatal physical activity and nutrient metabolism, and the professional nighttime monitoring ensures the stability of the neonatal internal environment and the continuity of growth and development. Although there was no significant difference in body weight and head circumference at 36 weeks of corrected gestational age, the continuous growth advantage before discharge suggested that the NIFCC model has a long-term positive effect on the physical growth of very preterm infants, which was worthy of further follow-up observation.

In terms of neurobehavioral development, the significantly higher NBO score in the study group reflects the effectiveness of the NIFCC model in promoting the neurobehavioral development of very preterm infants (10). Early identification of neurodevelopmental risks enabled timely intervention. NBO was characterized by family-centered approach, which enhances parental understanding of infant cues, thus supporting bonding and optimizing developmental outcomes (21). The multi-sensory stimulation in rehabilitation interventions, such as deep pressure, graded touch, and vestibular stimulation, combined with parent-child interactive games guided by therapists, could effectively activate the neonatal sensory-motor system, thus may promote the maturation of the nervous system (22). Meanwhile, the frequent parent-infant contact in daytime care can reduce the neonatal stress response and improve their behavioral regulation ability, which was an important reason for the improvement of neurobehavioral scores (23).

Notably, the incidence of BPD, IVH, LOS and other complications was comparable between the study group and the control group, which fully verified the clinical safety of this model. The key to this result was the rational design of the NIFCC model that balanced family participation and professional safety—infants return to the NICU for professional monitoring and nursing at night, which ensured the timely handling of abnormal conditions such as desaturation and apnea, and avoids the potential safety risks caused by insufficient parental care capacity at night. In addition, the standardized training for parents and the one-on-one guidance of therapists in rehabilitation interventions reduced the incidence of adverse events caused by improper operation, making the high participation of families and systematic rehabilitation intervention under the premise of safety (24).

Regarding the rehabilitation therapy implementation, the extremely significant difference in the implementation rate of various rehabilitation measures between the two groups might be the core factor for the better outcomes of the study group. The conventional care model in the control group only implemented a small number of rehabilitation measures such as swallowing dysfunction training, with low coverage and discontinuous intervention, which was difficult to form a synergistic effect on neonatal functional improvement (25). In contrast, the NIFCC model took individualized rehabilitation as an important component, with a dedicated physical therapist developing a personalized rehabilitation plan for each infant, and the high implementation rate of comprehensive rehabilitation measures formed a multi-dimensional intervention system for respiratory, motor, sensory and swallowing functions, which effectively solves the multiple functional deficits of very preterm infants and achieves the improvement of short-term outcomes.

Several limitations of this study should be acknowledged. First, the sample size was relatively small and the study is a single-center retrospective case-matched study, which may lead to selection bias and limit the generalizability of the results. Second, the follow-up period was limited to the in-hospital short-term outcomes before discharge, and the long-term effects of the NIFCC model on the neurodevelopmental, physical growth and long-term complication incidence of very preterm infants need to be further verified by long-term follow-up (26). Third, the study does not systematically assess the psychological outcomes of parents such as anxiety, depression and care confidence, and the impact of the NIFCC model on parental mental health and care capacity needs to be further explored. Finally, the implementation of the NIFCC model required dedicated rehabilitation staff, modified ward layout, and coordinated daytime-nighttime handover; thus, its feasibility in centers with limited personnel or space remains to be confirmed.

## Conclusions

This novel integrated family-centered care model was safe, feasible, and effective for VPIs. It significantly accelerated oxygen weaning and oral feeding establishment, improved anthropometric growth and neurobehavioral development at discharge. Future multi-center studies with long-term neurodevelopmental follow-up and comprehensive parental outcome assessments were warranted to further validate the effectiveness and generalizability of this model. If confirmed, the NIFCC model can serve as a promising strategy to optimize care quality and improve outcomes for preterm infants in NICUs worldwide.

## Data Availability

The raw data supporting the conclusions of this article will be made available by the authors, without undue reservation.
